# Early Reconstitution of Antibody Secreting Cells after Allogeneic Stem Cell Transplantation

**DOI:** 10.3390/jcm11010270

**Published:** 2022-01-05

**Authors:** Martina Hinterleitner, Clemens Hinterleitner, Elke Malenke, Birgit Federmann, Ursula Holzer, Martin Müller, Wolfgang A. Bethge, Stefan Wirths

**Affiliations:** 1 Department of Hematology, Oncology, Clinical Immunology and Rheumatology, University Hospital Tuebingen, 72076 Tuebingen, Germany; Martina.Hinterleitner@med.uni-tuebingen.de (M.H.); birgit.federmann@med.uni-tuebingen.de (B.F.); Martin.Mueller@krh.eu (M.M.); wolfgang.bethge@med.uni-tuebingen.de (W.A.B.); 2 Department of Medical Oncology & Pneumology (Internal Medicine VIII), University Hospital Tuebingen, 72076 Tuebingen, Germany; 3 DFG Cluster of Excellence 2180 ‘Image-Guided and Functional Instructed Tumor Therapy’ (IFIT), University of Tuebingen, 72076 Tuebingen, Germany; 4 Department of Pediatric Hematology and Oncology, University Hospital Tuebingen, 72076 Tuebingen, Germany; Elke.Malenke@med.uni-tuebingen.de (E.M.); Ursula.Holzer@med.uni-tuebingen.de (U.H.); 5 Department of Pathology and Neuropathology, University Hospital Tuebingen, 72076 Tuebingen, Germany; 6 Department of Hematology, Oncology and Immunology, Klinikum Region Hannover, KRH Klinikum Siloah, 30459 Hannover, Germany

**Keywords:** hematology, immune cells, reconstitution, allogenic stem cell transplantation

## Abstract

Immune cell reconstitution after stem cell transplantation is allocated over several stages. Whereas cells mediating innate immunity recover rapidly, adaptive immune cells, including T and B cells, recover slowly over several months. In this study we investigated kinetics and reconstitution of de novo B cell formation in patients receiving CD3 and CD19 depleted haploidentical stem cell transplantation with additional in vivo T cell depletion with monoclonal anti-CD3 antibody. This model enables a detailed in vivo evaluation of hierarchy and attribution of defined lymphocyte populations without skewing by mTOR- or NFAT-inhibitors. As expected CD3^+^ T cells and their subsets had delayed reconstitution (<100 cells/μL at day +90). Well defined CD19^+^ B lymphocytes of naïve and memory phenotype were detected at day +60. Remarkably, we observed a very early reconstitution of antibody-secreting cells (ASC) at day +14. These ASC carried the HLA-haplotype of the donor and secreted the isotypes IgM and IgA more prevalent than IgG. They correlated with a population of CD19^−^ CD27^−^ CD38^low/+^ CD138^−^ cells. Of note, reconstitution of this ASC occurred without detectable circulating T cells and before increase of BAFF or other B cell stimulating factors. In summary, we describe a rapid reconstitution of peripheral blood ASC after CD3 and CD19 depleted haploidentical stem cell transplantation, far preceding detection of naïve and memory type B cells. Incidence before T cell reconstitution and spontaneous secretion of immunoglobulins allocate these early ASC to innate immunity, eventually maintaining natural antibody levels.

## 1. Introduction

Immune cell reconstitution after hematopoietic stem cell transplantation (HSCT) has major influence on control of bacterial, viral and fungal infections, on graft-versus-host disease (GvHD) and on graft-versus-leukemia/lymphoma (GvL) effects [1,2]. In addition, it may give insight into fate decisions of hematopoietic stem cells in reconstitution of innate and adaptive immunity. Whereas innate immunity including granulocytes, monocytes or NK cells recover within weeks [3,4], recovery of adaptive immune cells, including CD4^+^, CD8^+^ T and B cells, takes up to 2 years [2,4]. T cell reconstitution after HSCT has been extensively studied [5,6,7]. Consecutive analysis of surface markers associated with B cell differentiation has identified transitional CD19^+^ CD24^high^ CD38^high^ B cells as one of the first CD19^+^ B cell subsets detected 6 to 8 weeks after HSCT [8]. Innate-like B cells compromise a heterogeneous group of unconventional B cells showing a T cell independent function, are involved in early innate immune responses, spontaneously secret immunoglobulins, exhibit a skewed VDJ-gene usage, and have been discussed as major source of natural poly- and autoreactive antibodies (NAbs) [9,10,11]. In mice, they are well characterized as B1 B cells. In humans, a minute subset of circulating CD19^+^ B cells with the above characteristics are detected, carrying a CD20^+^ CD27^+^ CD43^+^ CD70^−^ phenotype [12]. It is yet undefined whether recovery of lymphoid cells follow the same hierarchy of primary innate type followed by adaptive lymphocyte reconstitution.

In this study we consecutively analyzed kinetics and reconstitution of defined B cell populations in patients undergoing a T cell and B cell depleted haploidentical stem cell transplantation with additional in vivo T cell depletion with the monoclonal anti-CD3 antibody OKT3 [13,14]. This approach opens the possibility to analyze de novo B cell reconstitution in vivo without any influence of calcineurin inhibitors (CIs) or other immunosuppressive agents and without presence of preformed donor-derived B cells in the graft. Interestingly, as soon as 2 weeks after HHCT we observed reconstitution of spontaneously IgM, IgA and IgG secreting cells in all analyzed patients. These ASC were shown to be of donor origin and correlated with a yet undefined population of CD19^−^ CD27^−^ CD38^low/+^ CD138^−^ cells. These ASC developed independently of T cells, before transitional B cells and without detection of B cell stimulating factors. As they spontaneously secrete immunoglobulins, preferentially of IgM and IgA-type, they share characteristics of innate-like B cells and thus might be associated to innate rather than adaptive immunity.

## 2. Materials and Methods

### 2.1. Patients

Between 2008 and 2009, 10 consecutive patients (seven women and three men), enrolled in a prospective phase II study of haploidentical hematopoietic cell transplantation (HHCT) using CD3/CD19-depleted grafts (NCT00202917) [13], at the Department of Haematology, Oncology and Immunology, University Hospital Tuebingen (Germany) were analyzed in this study. Details on patient characteristics are given in Table S1. Three patients (one woman and two men) showing poor graft function, graft failure or sudden death on day +2 after HHCT were excluded. All patients signed an informed consent. In all patients PBMCs were collected at day 0 prior HHCT, as well as on day +7 (week 1), +14 (week 2), +21 (week 3), up to 36 weeks after HHCT. Freshly isolated PBMCs were analyzed at time point of sample collection. Serum samples were collected once per week and analyzed at the end of the observation period. This study was approved by the IRB (ethics committee of the Faculty of Medicine of the Eberhard Karls University Tuebingen) of the University Hospital Tuebingen and was conducted in accordance with the Declaration of Helsinki; reference number 21/2004.

### 2.2. Conditioning Regimen and Haploidentical Hematopoietic Cell Transplantation (HHCT)

All patients were treated in a prospective phase II study using CD3/CD19-depleted grafts (NCT00202917) as previously described [13,14]. In brief, patients received a reduced intensity conditioning (RIC) with fludarabine (150–200 mg/m^2^) day −8 to −4, thiotepa (10 mg/kg) day −3, melphalan (120 mg/m^2^) day −2 to −1 and OKT-3 (5 mg/day) day −5 to +14. CD3/CD19 depleted cryopreserved peripheral blood stem cells were given on day 0. In none of the patients granulocyte colony-stimulating factor (G-CSF) was used post HHCT. Patients received a short course of postgrafting immunosuppression with mycophenolate mofetil from day 0 to day +30. On day 15, patients were substituted with 15 g of IVIGs, devoid of IgA and IgM.

### 2.3. Flow-Cytometry Based Analysis of Immune Reconstitution and Chimerism

Hematopoietic donor cell chimerism was performed as previously described [15]. Immune reconstitution was monitored in peripheral blood mononuclear cells (PBMCs) by flow cytometry in between week 0–38. PBMCs were isolated by density gradient centrifugation using Biocoll cell separation solution (Biochrom, Berlin, Germany). Immune cells were stained using the directly fluorescence labeled mAbs CD138-PE (clone: MI15, BD Biosciences, Franklin Lakes, NJ, USA), CD138-PE (clone: MI15, BD Biosciences), CD38-PerCP (clone: HB-7, BD Biosciences), CD19-PacificBlue (clone: SJ25C1, BD Biosciences), CD27-FITC (clone: M-T271, BD Biosciences), IgA-APC (clone: 97924, R&D Sytems, Minneapolis, MN, USA), IgG-APC (clone: 97924, R&D Systems), IgM-APC (clone: IL/41, ThermoFisher), HLA-A2-APC (clone: BB7.2, ThermoFisher), HLA Class I B7-PE (clone: BB7.1, abcam, Cambridge, UK), HLA-Class1 BW6-PE (Miltenyi Biotec, Bergisch Gladbach, Germany) and HLA-Class1 B8-APC (Miltenyi Biotec). Dead cells were excluded from analysis by LIVE/DEAD™ Fixable Aqua Dead Cell Stain (ThermoFisher, Waltham, MA, USA). Measurements were performed using a FACS Canto II or FACS Fortessa (BD Biosciences) and data analyzed using the software FlowJo V10 (FlowJo LCC, Ashland, OR, USA).

### 2.4. ELISpot Assay to Detect IgM, IgG and IgG Antibody Secreting Cells

PBMCs from patients were isolated at defined time points and analyzed by modified enzyme-linked immunospot (ELISPOT) assay in duplicates. IgM, IgG and IgA ELISPOT assays in our study were performed as described previously [16,17]. In brief, 96-well plates (MAHA S4510, Millipore, Burlington, MA, USA) were coated overnight at 4 °C with antibodies against IgM, IgA or IgG (Sigma Aldrich). Plates were washed twice with PBS and blocked for 2 h at 37 °C with PBS/1% BSA before cells incubated overnight at 37 °C, 5% CO_2_ in humidified atmosphere. Plates were then washed four times with PBS/0.25% Tween-20. Detection solution (detection antibody diluted 1:2000 in PBS/1% BSA) was added for 2 h. Plates were washed four times with PBS/1% BSA prior adding streptavidin (1:400) for 1 h. Plates were washed twice with PBS/0.25% Tween-20 and twice with PBS. Spots were developed adding 100 µL/well of 3-amino-9-ethylcarbazole at 0.3 mg/mL diluted in 0.1 M sodium acetate pH 5.0 and 0.03% hydrogen peroxide for 5 min in the dark. After several washing steps with distilled water and overnight drying spots were counted with the ImmunoSpot Series 3B Analyzer and ImmunoSpot 3.0 software (CTL, Cleveland, OH, USA). To exclude unspecific binding a human IgM/IgG Double-Color ELISPOT (hIgMIgG-DCE-1M/2, ImmunoSpot^®^, Cleveland, OH, USA) assay kit was used according to the manufacturer’s instructions.

### 2.5. Detection of Immunoglobulin and Cytokine Levels Using Multiplex Immunoassay System

In order to detect and quantify immunoglobulin and cytokine levels of IL-1b, IL-2, IL-4, IL-5, IL-6, IL-7, IL-8, IL-10, IL-12, IL-13, IL-15, IL-17, G-CSF, GM-CSF, IFNγ, MCP-1 (MCAF), MIP-1β, TNFα, IgA, IgE, IgG and IgM in patients after HHCT, Bio-Plex Multiplex Immunoassay System (Bio-PlexTM Cytokine Assay and Bio-Plex ProTM Immunoglobulin Isotyping, Bio-Rad, Hercules, CA, USA) was performed according to the manufacturer’s instructions.

### 2.6. ELISA

Protein levels of BAFF were measured using a human BAFF ELISA kit (ab188391, abcam) according to the recommendations of the manufacturer. All concentrations are expressed as means ± SEM of triplicates.

### 2.7. Statistical Analysis

Student’s *t* test, Mann–Whitney U test, one-way ANOVA and Friedman’s test was used for continuous variables. If significant differences by ANOVA were found, group wise comparison was done (Tukey’s multiple comparison test). If significant differences were by Friedman’s test were found Dunn’s multiple comparisons test was used. Bars and error bars represent means of results and standard error of mean, respectively. All statistical tests were considered statistically significant when *p* was below 0.05. Statistical analysis was performed using GraphPadPrism (v.8.1.0).

## 3. Results

### 3.1. Early Increase of Serum Immunoglobulins and ASCs after HHCT with T and B Cell-Depleted Grafts

**Figure 1:**
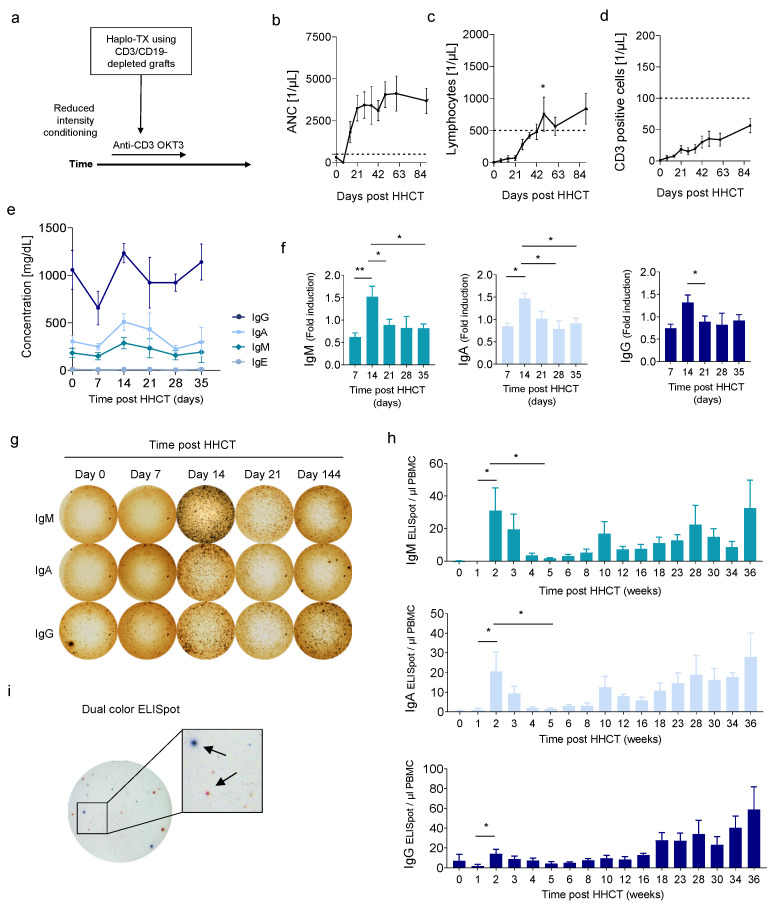
Immune cell reconstitution, detection of immunoglobulins and ASC after HHCT. (**a**) Schematic illustration of the study protocol (NCT00202917) (**b**) Reconstitution of absolute neutrophil count (ANC) after HHCT. Median on day + 14 (1815 cells/μL). (**c**) Reconstitution of lymphocytes after HHCT. Median on day + 14 (64 cells/μL). (**d**) Reconstitution of CD3 positive cells. Median on day + 14 (8 cells/μL). (**b**–**d**) Dotted line: clinically relevant lower threshold. (**e**) Serum level of IgG, IgA, IgM and IgE (mg/dL) after HHCT. (**f**) Fold induction (referring to day 0) of IgM, IgA and IgG in week 1–5 after HHCT. (**g**) Representative IgM, IgA and IgG B cell ELISpots on day 0, 7, 14, 21 and 144 after HHCT. (**h**) Quantification of IgM, IgA and IgG ELISpots/µL PBMC between week 0–35 after HHCT. (**i**) Dual color IgG, IgM B cell Immunospot. * < 0.05, ** < 0.01.

To investigate immune reconstitution after HHCT using CD3/CD19-depleted grafts we analyzed seven consecutive patients for a duration of 36 weeks (Figure 1a). Granulocyte engraftment (ANC > 500 cells/µL) was observed at day +10 (range day 9–13) (Figure 1b). Whereas total lymphocyte count increased significantly at day +49 (*p* = 0.02, Figure 1c) recovery of CD3^+^ T cells was shown to be delayed (<100 cells/μL at day +90, Figure 1d).

In order to analyze the role of ASC after HHCT with CD3/CD19-depleted grafts we performed longitudinal analysis of serum concentrations of IgM, IgA, IgG and IgE using Multiplex Immunoassay. Interestingly, in all patients we observed an unexpected peak of IgM, IgA and IgG level 14 days after HHCT (Figure 1e). IgE level, however, did not changed significantly. When Ig level were normalized to day 0, this peak was shown to be highly significant (Figure 1f). It is noteworthy that the increase of IgM, IgA and IgG 2 weeks after HHCT was only transiently observed, since at later time points (day ≥ 21) all three Ig level decreased again (Figure 1f). Increase of serum IgG may reflect substitution with IVIGs, that are devoid of IgM and IgA. To further investigate if the early increase of serum Ig might be associated with ASC, we took advantage of a modified ELISpot assay to detect IgM, IgA and IgG secreted by circulating PBMCs (Figure 1g).

Correlating with the above observation we detected a transient peak of IgM, IgA and IgG ELISpots/µL PBMC 14–21 days after HHCT (Figure 1h). Whereas all three Igs showed significant increased ELISpots/µL PBMC, IgM (31 ± 37.2) and IgA (20.5 ± 25.9) isotypes were more prevalent than IgG (14 ± 12). To exclude unspecific binding an IgM/IgG Double-Color ELISPOT was performed additionally (Figure 1i). In conclusion our findings suggest the existence of so far unexplored early ASC after HHCT secreting IgM, IgA and IgG.

### 3.2. Correlation of CD19^−^ CD27^−^ CD38^low/+^ CD138^−^ Cells with Early ASC after HHCT

**Figure 2:**
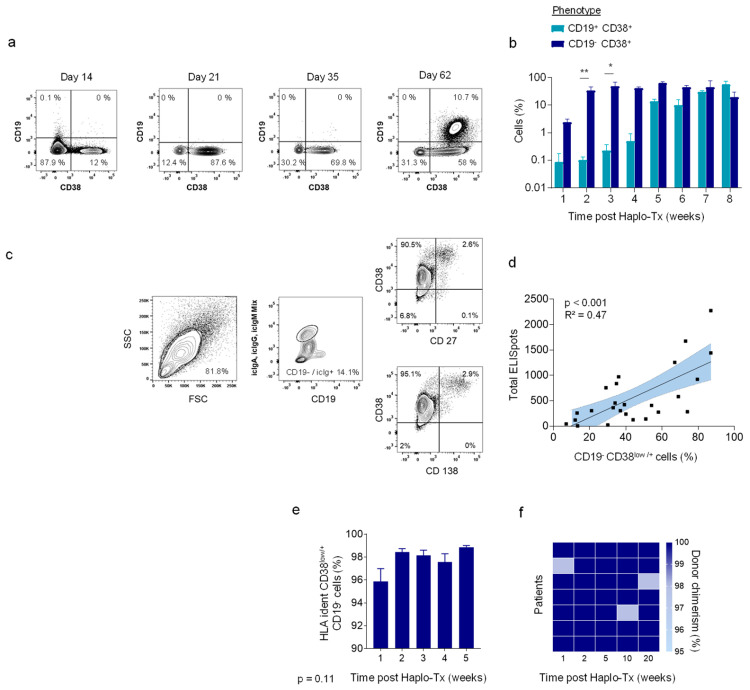
Reconstitution of B cells and antibody secreting cells (ASC). (**a**) Representative flow cytometry gating showing reconstitution of CD19 and CD38 positive immune cells. (**b**) Quantitative analysis of CD19^+^ CD38^+^ and CD19^−^ CD38^+^ immune cell populations in week 1–8 after HHCT. (**c**) Identification of immature IgM, IgA and IgG presenting CD38^low/+^ CD19^−^ CD27^−^ CD138^−^ cells two weeks after HHCT. (**d**) Linear regression analysis of total number of ELISpots (IgM, IgG and IgA) and frequency of CD38^low/+^ CD19^−^ cells in % of total PBMCs (*p* < 001, R^2^ = 0.47). (**e**) Frequency of CD38^low/+^ CD19^−^ cells presenting donor HLA phenotype (*p* = 0.11). (**f**) Donor chimerism of all patients in week 1, 2, 5, 10 and 20 after HHCT. * < 0.05, ** < 0.01, low/+ = low expression.

To further investigate the hypothesis of early, so far undefined ASC after HHCT we consequently monitored defined lymphocyte populations in all patients. Even if first naïve B cells were observed 5–6 weeks after HHCT we didn’t detect relevant levels of plasma cells during the first 8 weeks after HHCT (Figure S1). As observed previously, we identified engrafting of naive CD19^+^ CD27^−^ CD38^+^ B cells 6–8 weeks after HHCT (Figure 2a,b) [8]. In addition, we identified an early CD19^−^ CD38^low/+^ cell population (Figure 2a). Of note, this population was already detectable 7–14 days after HHCT (Figure 2b). Further characterization showed high amounts of intracellular immunoglobulins, suggesting that these cells might be causal for the early increase of serum Ig. About 3% of these intracellular Ig^high^ cells mirrored the phenotype of well characterized long-lived plasma cells (CD27^+^ CD38^++^ CD138^+^). However, the vast majority of intracellular Ig^high^ cells revealed a so far undefined phenotype of CD19^−^ CD27^−^ CD38^low/+^ CD138^−^ (Figure 2c). The finding, that the proportion of CD19^−^ CD38^low/+^ cells positively correlated with the total number of detected ELISpots further supports the hypothesis that this so far undefined population may secret relevant amounts of IgM, IgA and IgG (Figure 2d). To exclude that the observed population of CD19^−^ CD27^−^ CD38^low/+^ CD138^−^ cells are of are of patient origin, we performed FACS analysis of the HLA-haplotypes which confirmed the donor origin in all patients (Figure 2e). Moreover, analysis of donor–recipient chimerism at week 2 revealed that >99% of recipient cells carried donor alleles (Figure 2f).

### 3.3. Cytokine Levels during Immune Cell Reconstitution

**Figure 3:**
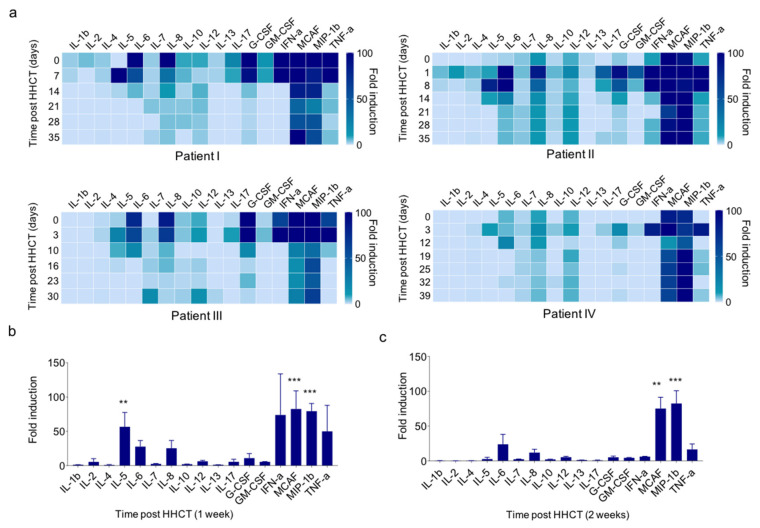
Serum cytokine levels during immune cell reconstitution after HHCT. (**a**) Serum cytokine level of IL-1b, IL-2, IL-4, IL-7, IL-10, IL-12, IL-6, IL-8, IFNα, MCAF, MIP-1b and TNFα in four representative patients in-between 0–39 days after HHCT. (**b**) Quantitative analysis of cytokine level one week and two weeks (**c**) after HHCT. ** < 0.01, *** < 0.001.

B cell reconstitution and maturation after HSCT and HHCT is a complex multistep process influenced by several patient-, donor-, treatment- and disease-related factors orchestrated via several cytokines [2,18,19]. In order to identify cytokines associated with the development of ASC in the described setting we characterized cytokine composition during the first month after HHCT (Figure 3a). Whereas no significant levels of IL-1b, IL-2, IL-4, IL-7, IL-10, IL-12 and IL-13 was observed, we detected a significant increase of IL-5 level in all patients 1 week after HHCT (Figure 3b or Figure S2a). In addition, we observed increased levels of inflammatory cytokines including IL-6, IL-8, IFNα, MCAF, MIP-1b and TNFα (Figure 3a,b). While the most of these cytokine levels dropped over time, elevated MCAF and MIP-1b levels persisted during the first month after HHCT (Figure 3a). Since BAFF plays a significant role in B cell maturation and has been shown to enhance survival of plasmablasts derived from human memory B cells, we additionally investigated serum concentrations of BAFF in our patient cohort [20]. Of note, during the first 10 weeks after HHCT we did not observe relevant level of BAFF (Figure S2b). Whereas we observed a significant correlation of BAFF plasma levels or CD3^+^ T cells and plasma cells in our cohort, development of early ASC were found to be independent of these two factors (Figure S2c,d).

## 4. Discussion

Whereas cells of the innate immune system including monocytes or NK cells can already be detected 2 weeks after HSCT, populations of ASC are commonly described after six to twelve months [1,19]. However, first emerging B cell subsets can be detected already 6–8 weeks after HSCT and are mainly characterized as transitional or mature naïve CD19^+^ CD27^−^ CD38^high^ B cells [8]. Innate-like B cells in mice are well characterized as B1 B cells, that are spontaneously secreting Ig, are readily activated by innate signaling without any T-cell help, use of a skewed VDJ-repertoire and are thought to provide a repertoire of poly- and autoreactive natural immunoglobulins [9,10,11]. In humans, only a minute subset of circulating CD19^+^ B cells with the above characteristics are identified as CD20^+^ CD27^+^ CD43^+^ CD70neg [12]. In this study we took advantage of a CD3 and CD19 depleted haploidentical stem cell transplantation accompanied by an in vivo T cell depletion with a monoclonal anti-CD3 antibody [13,14]. It opens up the unique possibility to consecutively analyze the de novo development and reconstitution of B cell function including ASC in vivo. In accordance with previous data we observed engrafting transitional B cells six-eight weeks after HHCT [1,8,19]. We identified a not yet characterized population of CD19^−^ CD27^−^ CD38^low/+^ CD138^−^ cells with high intracellular levels of either IgM, IgA or IgG. These cells spontaneously secreted preferentially IgM and IgA, and to a lesser extent IgG. As these cells are negative for CD19 they do not meet the criteria for conventional immature B cells [21]. Of note, the substitution of polyvalent IgG might influence the observed peak of IgG at day + 14 in our setting. However, we additionally observed a peak of IgM and IgA and validated this observation in our ELISpot assay. Even if the existence of a small fraction of plasma cells might be a potential source of bias in our setting, early ASC developed independently from T cells and cytokines associated with the development of B cells and well characterized plasmablasts or long-lived plasma cells. Thus, they might represent a so far undescribed population of innate-like B cells. This hypothesis is further supported by the finding, that reconstitution of these cells was accompanied by an increase of IL-5, which has been described to be involved in a T cell-independent development of innate-like B cells and consecutive Ig production [22]. In addition, we observed a profound increase of IL-6, macrophage inflammatory protein-1 beta (MIP-1b) and monocyte chemotactic and activating factor (MCAF). Since these cytokines are related to macrophage homeostasis on the one hand, and macrophages has been described to share several similarities with innate B cells in terms of their developmental origins and effector functions [23], a functional crosstalk of these cytokines and our observed early ASC population might be speculated. However, further data are needed to investigate such a hypothesis. Our finding that the identified CD19^−^ CD27^−^ CD38^low/+^ CD138^−^ cells were of donor origin further supports the assumption of a preferential engraftment of innate-like B cells after HCT. Even if our setting opens up the possibility to study B cell reconstitution in vivo without any influence of CIs or other immunosuppressive agents and without presence of preformed donor-derived B cells in the graft, our observation might be specific to this type of HCT. Additional data in varying HCT settings are warranted to further investigate this putatively innate B cell type. Nevertheless, the used setting of HHCT in our study might be a suitable model and give further insights in immune cell reconstitution.

## Data Availability

The data that support the findings of this study are available from the corresponding author upon reasonable request.

## Supplementary Materials

The following are available online at https://www.mdpi.com/article/10.3390/jcm11010270/s1, Table S1: Patients characteristics. Figure S1: Fold induction of IL-5 and BAFF level after HHCT; Figure S2: (a) Fold induction (referring to 5 healthy donors) of IL-5 1-5 weeks after HHCT. (b) Fold induction and serum concentration (ng) of BAFF after HHCT. (c) Correlation of BAFF or CD3 positive cells and plasma cells after HHCT. (d) Correlation of BAFF or CD3 positive cells and early ASC after HHCT.
