# Supplementary material for: Early Reconstitution of Antibody Secreting Cells after Allogeneic Stem Cell Transplantation
Source: J Clin Med. 2022 Jan 5;11(1):270. doi: 10.3390/jcm11010270 (PMC8745805; doi:10.3390/jcm11010270)
Supplement: Supplementary file 1 [file jcm-11-00270-s001.zip › jcm-1503542-supplementary.pdf]

Supplementary Figures and Tables

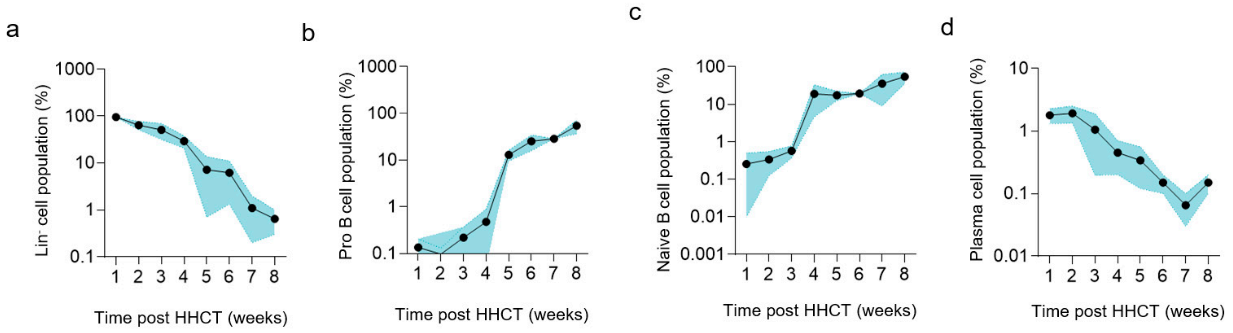

Figure S1: Immune reconstitution of Lin- (**a**), pro B cells (**b**), naïve B cells (**c**) and plasma cells (**d**) after HHCT. Lin = Lineage, HHCT = Haploidentical Hematopoietic Cell Transplantation,

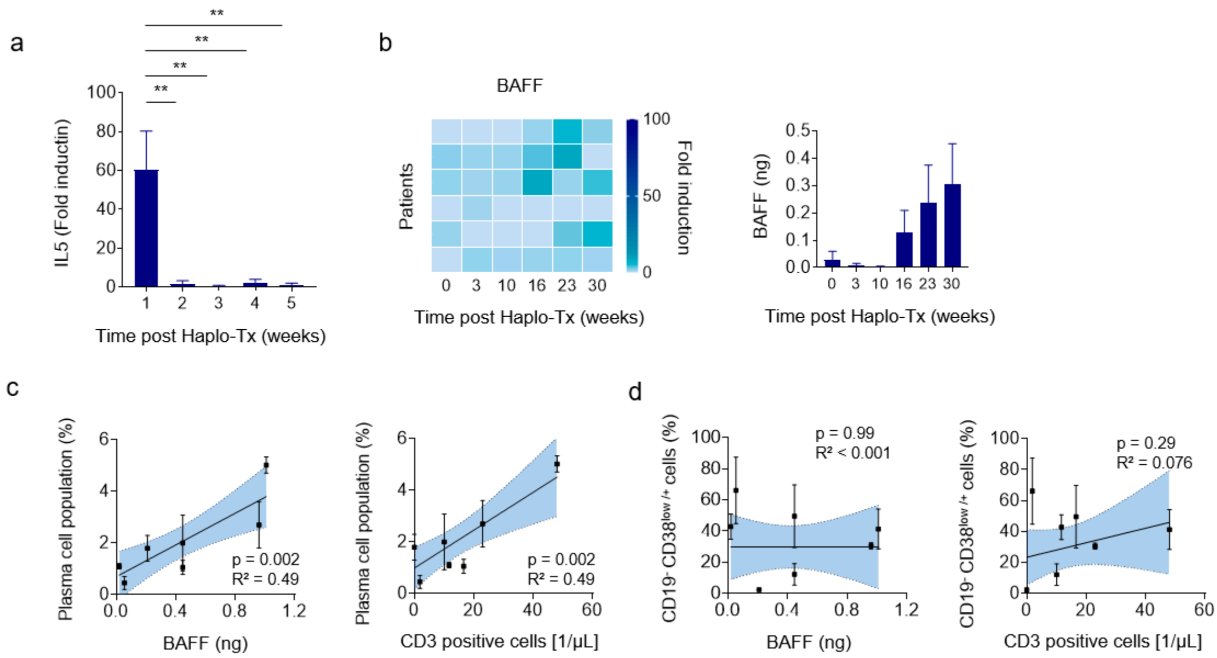

Figure S2: (**a**) Fold induction (referring to 5 healthy donors) of IL-5 1-5 weeks after HHCT. (**b**) Fold induction and serum concentration (ng) of BAFF after HHCT. (**c**) Correlation of BAFF or CD3 positive cells and plasma cells after HHCT. (**d**) Correlation of BAFF or CD3 positive cells and early ASC after HHCT. IL = Interleukin, BAFF = B-cell activating factor, CD = cluster of differentiation, ** < 0.01.

Table S1: Patients characteristics

Patient characteristics	Total (n=10)
**Gender**	
male sex, n (%)	3 (30)
**Age**	
Age at study inclusion in years, mean–yr.(95%CI)	42.6 ± 10.34 (25 to 57)
**Diagnosis, n (%)**	
Acute myeloid leukemia (AML)	9 (90)
Multiple myeloma (MM)	1 (10)
**Stage at HCT, n (%)**	
Complete Remission (CR)	3 (30)
Partial Remission (PR)	7 (70)
**Engraftment**	
ANC >500 cells/µl (days)	10 (9-13)
**Acute GVHD, n (%)**	4 (40)
Grade I°	2 (20)
Grade II°	1 (10)
Grade III°	1 (10)
Grade IV°	0
**Relapse, n (%)**	6 (60)
Patients still alive, n (%)	4 (40)

CI=confidence interval, HCT= Hematopoietic Cell Transplantation, ANC= absolute neutrophil count, GVHD= Graft-versus-host disease, n=number
